# Adult Congenital Heart Disease: Report from a Public Reference Hospital in Northeastern Brazil

**DOI:** 10.21470/1678-9741-2023-0039

**Published:** 2023-09-19

**Authors:** Maria Suely Bezerra Diogenes, Acrísio Sales Valente, Hermano Alexandre Lima Rocha

**Affiliations:** 1 Hospital de Messejana Dr. Carlos Alberto Studart Gomes, Fortaleza, Ceará, Brazil; 2 Department of Public Health, Faculdade de Medicina, Universidade Federal do Ceará, Fortaleza, Ceará, Brazil

**Keywords:** Congenital Heart Defects, Atrial Septal Defect 5, Tetralogy of Fallot, Cardiac Surgical Procedures, Reoperation

## Abstract

**Introduction:**

The increasing worldwide number of adults with congenital heart disease (CHD) demands greater attention from health professionals. The purpose of this report is to describe the clinical demographic profile, frequency, and invasive treatment status of adults with CHD in a public reference hospital in northeastern Brazil.

**Methods:**

This is a retrospective cross-sectional study including 704 patients attended between August 2016 and August 2020. Data were collected from virtual database.

**Results:**

Patients’ age varied from 17 to 81 years (mean 32±14; median 27 years); 294 (41.8%) patients were male, and 410 (58,2%) were female; 230 (32,7%) had diagnosis from age 18 and up. Cardiac complexity categories were “simple defects” (134 [19%] patients), “moderate complexity” (503 [71.5%]), and “great complexity” (67 [9.5%]). Atrial septal defect (ASD) was diagnosed in 216 (30.7%) patients, ventricular septal defect (VSD) in 101 (14.3%), tetralogy of Fallot in 93 (13.2%), and other CHD in 294 (41.8%). New York Heart Association (NYHA) functional classes were I (401 [57%]), II (203 [28.8%]), III (76 [10.8%]), and IV (24 [3.4%]). Complications were arrhythmias (173 [24%]) and severe pulmonary hypertension (69 [9.8%]). Invasive treatments were corrective surgery (364 (51.6%]), reoperation (28 [4.0%]), palliation (11 [1.6%]), interventional catheterization (12 [1.7%]), surgery plus interventional catheterization (5 [0.7%]), and preoperation (91 [12.9%]). Treatment was not required in 102 (14,5%) patients, and 91 (12.9%) were inoperable.

**Conclusion:**

The leading diagnosis was ASD. Frequency of unrepaired patients was high, mainly ASD, due to late diagnosis, which favored complications and denotes a matter of great concern.

## INTRODUCTION

The growing worldwide adult population with congenital heart disease (CHD) is a consequence of the survival of children with heart defects successfully treated, especially the ones with complex defects, as the cyanotic group. This outcome is a result of the advance in new diagnostic methods and invasive treatment, particularly surgery^[1,2,3,4,5,6,7]^. Although more than 90% of children with CHD reach adulthood, the exact prevalence of the adult population is unknown^[8,9]^. This expansion of adult congenital cardiacs, already foreseen decades ago, requires better profile evaluation and action from health professionals once there can be clinical and hemodynamic deterioration of patients left with significant residual lesions of previous invasive treatment^[10,11,12]^.

In Brazil, publication is scarce, so little is known about the profile and frequency of adults with CHD^[13,14,15,16,17]^. It is possible that many patients repaired in childhood are not receiving any specialized assistance and many others did not undergo invasive treatment. Consequently, they may be evolving with complications like arrhythmias, heart failure, and pulmonary hypertension. Some patients could even be in inoperable situation resulting in increased morbimortality and gradually transforming this issue into a public health problem.

Since the clinical demographic profile, invasive treatment status, and frequency of adults with CHD are unknown in northeastern Brazil, the aim of the present paper is to outline these features in a cohort attended in a public reference facility.

## METHODS

An observational cross-sectional study with retrospective cohort was performed comprising 704 Brazilians with CHD, age ranging from 17 to 81 years, attended at the outpatient clinic of a reference public hospital in the cardiopulmonary field, in the city of Fortaleza (Ceará, Northeast Brazil). Patients’ clinical demographic information was obtained from a virtual database registered in a Microsoft Excel Program document collected during medical consultations in the period between August 2016 and August 2020 by the authors of the present paper. Physical medical records were reviewed whenever necessary. Patients were referred from pediatric cardiology and adult cardiology outpatient clinics of the hospital where the research was undertaken. Inclusion criteria were: patients born in Brazil, aged 17 years and up with CHD. Although most of the patients were adults, 17-year-old teenagers were also included since this age is considered a borderline between teenage and adulthood. According to Brazilian law, adulthood begins at 18 years^[18]^. Exclusion criteria were: foreigners, teenagers under 17 years of age, patients with concomitant Marfan Syndrome, and those with both CHD and rheumatic heart disease.

All patients had definite diagnosis, complemented by electrocardiogram (ECG), chest X-ray in the posterior-anterior view, and bidimensional echocardiogram with color flow Doppler, and other exams according with their needs.

The following variables were analysed: age; gender; origin (Fortaleza and metropolitan area, countryside, other states in the Northeast); diagnosis of CHD; age at diagnosis (under 18 years, 18 years and up); clinical presentation according to heart defect (acyanotic, cyanotic); heart failure clinical functional class (FC), classified into I, II, III, and IV, according to the New York Heart Association (NYHA); pulmonary arterial hypertension (PAH) defined according to European guidelines^[19]^; arrhythmias registered in standard ECG or Holter monitoring; and treatment status (surgical treatment [patients who underwent corrective or palliative surgery; patients were called reoperated if they had undergone one or more than one surgical intervention for correction of residual lesions later after corrective surgery]; interventional cardiac catheterization; combined surgical and interventional catheterization; unrepaired [this category included three types of patients — the ones awaiting surgical treatment, or preoperative status; patients with small defects who didn’t need any treatment; and patients in inoperable situation according to the criteria used for invasive treatment]).

Patients diagnosed at 18 years of age and up were considered having late diagnosis.

When two or more congenital heart defects were present, main diagnosis was considered the one with greater pathophysiological impact.

According to CHD complexity based on the anatomical classification of the American Heart Association/American College of Cardiology (or AHA/ACC)^[6]^, patients were classified into three categories:

- Category I – Simple Defects: isolated small defects — *ostium secundum* atrial septal defect (ASD), ventricular septal defect (VSD) —; mild isolated pulmonic stenosis; repaired conditions with no significant residual lesion, shunt, or chamber enlargement — ligated or occluded patent *ductus arteriosus* (PDA), repaired ASD, *sinus venosus* defect, and VSD.
- Category II – Moderate Complexity Defects: unrepaired moderate or large heart defects, repaired conditions with significant residual lesions — moderate and large unrepaired *secundum* ASD and PDA, VSD with associated abnormality and/or moderate or greater shunt, *sinus venosus* defect, partial or complete atrioventricular septal defect, moderate or severe pulmonic stenosis or regurgitation, infundibular right ventricular outflow obstruction, peripheral pulmonary stenosis (PS), congenital aortic valve disease, subvalvar aortic stenosis (excluding hypertrophic cardiomyopathy), supravalvar aortic stenosis, coarctation of the aorta, congenital mitral valve disease, Ebstein anomaly, tricuspid valve dysplasia, partial or total anomalous venous connection, straddling atrioventricular valve, anomalous coronary artery arising from the pulmonary artery, anomalous aortic origin of a coronary artery from the opposite sinus, aorto-left ventricular fistula, *sinus* of Valsava fistula/aneurysm, repaired tetralogy of Fallot.
- Category III – Great Complexity Defects (complex heart disease): all forms of repaired, palliated, or unrepaired cyanotic CHD; congenitally corrected transposition of the great arteries (ccTGA).

### Statistical Analysis

Data analysis was performed using software IBM Corp Released 2015, IBM SPSS Statistics for Windows, version 23.0, Armonk, NY: IBM Corp. Descriptive analysis was used and expressed in absolute and percentual (%) numbers for categorical variables, with their respective inferior limit (IL) and superior limit (SL) of the 95% confidence interval (CI). For numerical variables, mean and standard deviation values were used for continuous variables with normal distribution. For continuous variables without normal distribution, median and interquartile interval was applied. Chi-square test of Pearson, a non-parametric test, was used for comparison between categorical variables. Results were considered significant when *P*-value < 0.05.

The project of the present paper received approval of the Ethical Committee via “Plataforma Brasil”: “Certificado de Apresentação de Apreciação Ética (CAAE)” number 48865121.4.0000.5039.

## RESULTS

Clinical demographic profile of adult patients with CHD is displayed in Tables 1, 2, 3. Frequency of CHD is shown in Tables 4 and 5.

**Table 1:** Number of adult patients with congenital heart disease according to age range.

Age range (years)	N (%)	95% CI	
		Inferior CL	Superior CL
17 – 20	151 (21.4)	18.8%	24.9%
21 – 30	270 (38.4)	34.7%	41.9%
31 – 40	119 (16.9)	14.2%	19.7%
41 – 50	77 (10.9)	8.8%	13.4%
51 – 60	56 (8.0)	6.1%	10.1%
61 – 70	25 (3.6)	2.4%	5.2%
71 – 80	5 (0.7)	0.3%	1.6%
81 – 90	1 (0.1)	0.0%	0.7%
Total	704 (100)		

CI=confidence interval; CL=confidence limit

**Table 2:** Clinical demographic profile of adult patients with congenital heart disease.

Age (years)	Mean (SD)	Median
		IQI: 25% 50% 75%
	32 (±14)	21 27 38
	**N (%)**	**95% CI: inferior CL/superior CL**
Gender		
Female	410 (58.2)	54.6%/61.8%
Male	294 (41.8)	38.2%/45.4%
Total	704 (100)	
Origin		
Fortaleza and metropolitan area	347 (49.3)	45.7%/53.1%
Countryside	347 (49.3)	45.7%/53.1%
Other states	10 (1.4)	0.7%/2.5%
Age at diagnosis		
Under 18 years	474 (67.3)	63.8%/70.7%
18 years and up	230 (32.7)	29.3%/36.2%
Type of CHD		
Acyanotic	564 (80.1)	77.0%/82.9%
Cyanotic	140 (19.9)	17.1%/23.0%
Category/complexity		
I: simple defects	134 (19.0)	16.3%/22.1%
II: moderate complexity	503 (71.5)	68.8%/74.7%
III: great complexity	67 (9.5)	7.5%/11.9%

CHD=congenital heart disease; CI=confidence interval; CL=confidence limit; IQI=interquartile interval; SD=standard deviation

**Table 3:** Clinical profile of adult patients with congenital heart disease (N=704).

	N (%)	95% CI: inferior CL/superior CL
Functional class		
I	401 (57.0)	53.3%/60.6%
II	203 (28.8)	25.6%/32.3%
III	76 (10.8)	8.7%/13.2%
IV	24 (3.4)	2.3%/4.9%
Pulmonary hypertension		
Absent	589 (83.8)	80.9%/86.4%
Present: mild/moderate	45 (6.4)	4.8%/8.4%
Present: severe	69 (9.8)	7.8%/12.2%
Arrhythmias		
Absent	531 (76.0)	72.7%/79%
Present	173 (24.0)	
Types of arrhythmia		
AF or flutter	55 (7.8)	6.0%/10.0%
Other supraventricular	59 (8.4)	6.5%/10.6%
NSVT	5.0 (0.7)	0.3%/1.5%
Other ventricular	12 (1.7)	0.9%/2.9%
Supraventricular + ventricular	14 (2.0)	1.1%/3.2%
Bradycardia	28 (4.0)	2.7%/5.6%

AF=atrial fibrillation; CI=confidence interval; CL=confidence limit; NSVT=non-sustained ventricular tachycardia

**Table 4:** Most frequent congenital heart disease in adult patients according to diagnosis.

CHD		N=577/704	
	Subtype	N (%)	95% CI: inferior CL/superior CL
			
ASD	ASD OS	178 (25.2)	22.2%/28.6%
	ASD SV	23 (3.3)	2.1%/4.8%
	ASD OP	15 (2.1)	1.3%/3.4%
VSD		101 (14.3)	11.4%/17.9%
Tetralogy of Fallot		93 (13.2)	10.9%/15.9%
Shunt with PS		35 (5.0)	3.5%/6.8%
PS			
	Valvar	23 (3.3)	2.1%/4.8
	Subvalvar	05 (0.7)	0.3%/1.5%
	Supravalvar	03 (0.4)	0.1%/1.1%
Aortic stenosis			
	Subvalvar	16 (2.3)	1.4%/3.6%
	Valvar	10 (1.4)	0.7%/2.5%
	Supravalvar	03 (0.4)	0.1%/1.1%
Aortic coarctation		25 (3.6)	2.4%/5.1%
PDA		24 (3.4)	2.3%/4.9%
Ebstein anomaly		23 (3.2)	2.1%/4.8%

ASD=atrial septal defect; CHD=congenital heart disease; CI=confidence interval; CL=confidence limit; OP=*ostium primum*; OS=*ostium secundum*; PDA=patent *ductus arteriosus*; PS=pulmonary stenosis; SV=*sinus venosus*; VSD=ventricular septal defect

**Table 5:** Less frequent congenital defects in adult patients according to diagnosis.

Congenital heart disease	N=127/704	
	N (%)	95% CI: inferior CL/superior CL
Association of 2 or more shunts	13 (1.8)	1%/3%
ccTGA	11 (1.5)	0.9%/2.6%
CAVC defect	09 (1.3)	0.6%/2.3%
TGA	09 (1.3)	0.6%/2.3%
PA with VSD	09 (1.3)	0.6%/2.3%
DORV	08 (1.1)	0.5%/2.1%
Single ventricle	07 (1.0)	0.4%/1.9%
Bicuspid aortic valve with AR	07 (1.0)	0.4%/1.9%
Tricuspid atresia	06 (0.9)	0.4%/1.7%
Tricuspid valve dysplasia	06 (0.9)	0.4%/1.7%
Coronary anomaly	06 (0.9)	0.4%/1.7%
Coronary and aortic fistulae	06 (0.9)	0.4%/1.7%
PAPVC	03 (0.4)	0.1%/1.1%
Single atrium	02 (0.3)	0.1%/0.9%
TAPVC	02 (0.3)	0.1%/0.9%
Heterotaxy syndrome	01 (0.1)	0.0%/0.7%
Other defects	22 (3.1)	2.0%/4.6%

AR=aortic regurgitation; CAVC=complete atrioventricular canal; ccTGA=congenitally corrected transposition of the great arteries; CI=confidence interval; CL=confidence limit; DORV=double outlet right ventricle; PA=pulmonary atresia; PAPVC=partial anomalous pulmonary venous connection; TAPVC=total anomalous pulmonary venous connection; TGA=transposition of the great arteries; VSD=ventricular septal defect

Most acyanotic heart defects were isolated defects but it was observed association of two or three defects with the same pathophysiology of equal magnitude, like shunt lesions, or defects of different pathophysiology but with equal magnitude, like PS and a shunt lesion.

Among patients with ASD, 148 (68.5%) had late diagnosis and 68 (31.5%) were diagnosed under 18 years of age. Both groups were compared (*P*-value < 0.0001). The most frequent type of ASD was *ostium secundum* (*P*-value < 0.0001). Most of the patients were women (155 – 71.7%) and were predominantly in NYHA FC I (*P*-value < 0.0001). Fifty-six (25.9%) patients had supraventricular arrhythmia, and among these, 37 (17.1%) had either atrial fibrillation or atrial flutter.

Most patients with VSD were diagnosed under 18 years of age (87 patients – 86.1%). Fifty-three (52.5%) had small defects and did not need repair, 32 (31.7%) had been repaired in childhood, and only three (3%) patients were awaiting surgery. One patient operated in adulthood was in atrial flutter. All patients with cyanotic heart disease were diagnosed in infancy and childhood. Among patients with tetralogy of Fallot, all had been repaired, except five patients, and this occurred because of parents’ choice. There were 53 (57.0%) in NYHA FC I, 34 (36.5%) in FC II, four (4.3%) in FC III, and two (2.2%) in FC IV. Seventeen patients (18.3%) were awaiting reoperation for pulmonary valve replacement due to severe residual pulmonary regurgitation.

Among patients with ccTGA, only two didn’t have any associated defect. The oldest one was a 54-year-old woman who was evolving in NYHA FC II, with mild systemic morphological right ventricular dysfunction, and sustaining sinus rhythm. She was one of the four patients who had late diagnosis. Two patients were evolving with episodes of sustained supraventricular tachycardia: one of them was awaiting ASD repair and the other didn’t have any associated defect. Five patients had dysfunctional systemic morphological right ventricle. In the overall, six patients had undergone cardiac surgical procedures: four patients repaired associated defects, one patient had a double-switch procedure in another reference center in São Paulo (Brazil), and another had a palliative modified Blalock-Taussig shunt in childhood for severe PS. The latter patient was one of the two patients who had complex anatomy, and criss-cross heart was suspected. Among operated patients, two of them had a pacemaker implantation postoperatively for 2nd degree atrioventricular block Mobitz II.

Patients with the severe form of PAH were inoperable (Table 3). There were 55 patients with a previous left-to-right shunt lesion who progressed to Eisenmenger syndrome (inverted shunt): ASD (34), VSD (12), complete atrioventricular canal defect (seven), and PDA (two). The remaining patients with severe PAH had transposition of the great arteries (three), pulmonary atresia with VSD (two), double outlet right ventricle (one), total anomalous pulmonary venous connection (one), single atrium (one), and other cyanotic heart defects (six). Patients with ASD, single atrium, and PDA were all diagnosed beyond 18 years of age.

Considering invasive treatment status of the cohort (Table 6), 182 (25.8% – 95% CI IL=22.2%, SL=28.6%) unrepaired patients were either awaiting repair (preoperation) or were inoperable, and this was a consequence of late diagnosis, that is, those diagnosed at 18 years of age and up. Most of the patients classified as preoperative status had ASD (55 patients).

**Table 6:** Treatment status and types of invasive intervention in adults with congenital heart disease.

Treatment (status/types)	N (%)	95% CI: inferior CL/superior CL	
Preoperative	91 (12.9)	10.6%/15.6%	
Not required	102 (14.5)	12.0%/17.2%	
Inoperable	91 (12.9)	10.6%/15.6%	
Interventional catheterization	12 (1.7)	1.0%/3.0%	
Surgery + interventional catheterization	05 (0.7)	0.3%/1.5%	
Corrective surgery	352 (50.0)	46.3%/ 53.7%	
Other surgeries			Type of CHD
Fontan	8 (1.1)	0.5%/2.1%	TA, SV
Senning	3 (0.4)	0.1%/1.1%	TGA
Jatene	1 (0.1)	0.0%/0.7%	TGA
Palliative surgery			
MBTS	4 (0.6)	0.2%/1.3%	PA + VSD, PS, PA
BH	3 (0.4)	0.1%/1.1%	TGA
PA B	2 (0.3)	0.1%/0.9%	VSD, DORV
Bidirectional Glenn	2 (0.3)	0.1%/0.9%	SV
Reoperation	28 (4.0)	2.7%/5.6%	ToF
Total	704 (100)		

BH=Blalock-Hanlon; CI=confidence interval; CL=confidence limit; MBTS=modified Blalock-Taussig shunt; PAB=pulmonary artery banding

Interventional catheterization was applied in patients with ASD, PS, and PDA. Combined surgery plus interventional catheterization was applied in patients with coarctation of the aorta with significant residual coarctation.

Twenty-two patients with late diagnosis were following the natural history of the heart defect and were inoperable as a consequence of advanced heart failure. Half of them were ASD patients and over 60 years of age. The oldest was an 81-year-old woman with *sinus venosus* ASD. She was in atrial fibrillation and heart failure NYHA FC IV.

## DISCUSSION

Global view of adults with CHD invasively treated in infancy and childhood reveals a new reality in opposition to five decades ago: the progressive and rapidly rising number of adult patients with residual lesions from previous corrective surgical procedures, predominantly survivals with tetralogy of Fallot and complex heart defects. Many of this new generation of postoperative adult patients will evolve with future need for invasive reintervention^[1,2,3,4,5,6,7,12]^. In Brazil, in addition to the need to cope with the new reality, old challenges like unrepaired patients due to late diagnosis and related complications still represent great concern^[14,15,16,17]^.

Clinical demographic profile of the cohort studied herein showed significantly higher frequency of young adults, mainly women, similar to the findings observed in the southeastern Brazilian city of Ribeirão Preto^[16]^ and in the Asian country of Taiwan^[4]^. When analysing patients’ origin, there was no difference between Fortaleza, including metropolitan area, and other towns.

Most defects belonged to the moderate complexity category, differently from the findings by Amaral et al.^[16]^, in which predominated defects belonging to the low complexity category. Acyanotic CHD was significantly more frequent than cyanotic heart disease. In the overall, although most patients with acyanotic defects were diagnosed under 18 years of age, late diagnosis was a significant finding, especially in patients with ASD.

The most frequent CHD was ASD and the *ostium secundum* type significantly outnumbered other types. Although the number of unrepaired patients was high, in most of them invasive treatment was still feasible. The second most frequent CHD was VSD, mostly represented by a small defect which didn’t need any treatment. The majority of patients had early diagnosis and nearly all the ones who needed surgery had been repaired in childhood, the opposite to that observed in patients with ASD. Literature highlights septal defects, specially *ostium secundum* ASD and VSD as the most frequent CHD in adults^[2,3,4,14,15,16]^.

Tetralogy of Fallot was the third most frequent CHD and nearly all patients had been repaired. Literature considers it the most frequent operated cyanotic heart disease that survives childhood^[2,4,12]^. In the report by Ruiz et al.^[20]^, tetralogy of Fallot made up the greater part of the cohort. In the present study, severe pulmonary valve regurgitation was the residual lesion which led to reintervention in a considerable number of patients. These findings are in accord with the literature and call attention for the high incidence of adult patients with residual pulmonary regurgitation and increased morbidity as they reach middle age^[6,7,21]^.

Congenital heart defects of great complexity were the least frequent, and most of them had been totally corrected in childhood. Only a few patients were living with a palliative surgery. ccTGA slightly outnumbered other complex defects followed by other conotruncal anomalies. Almost half of the patients with ccTGA were evolving with systemic right ventricular dysfunction. Recently, a review article published by Amaral et al.^[22]^ emphasized the need for early diagnosis of this entity because of the implications concerning the systemic right ventricle for patients generally evolve with no symptoms in childhood and could reach elderly age, unrecognized.

Most patients analysed in the present paper were in NYHA FC I followed by FC II for heart failure. However, not a negligible number of patients were in FC III and FC IV. It is possible that the ones with advanced heart failure were unrepaired patients who evolved with complications and were not suitable for repair anymore, as the ones with Eisenmenger syndrome and those who were following the natural history of the disease. There were also the ones who had residual lesions from previous repairs. According to the literature, residual lesions and Eisenmenger syndrome are among the main causes of heart failure in adults with CHD^[5,6,7,23,24]^. Arrhythmias were the most frequent complications, particularly supraventricular arrhythmias like atrial flutter and fibrillation, and outnumbered the reports by Wu et al.^[4]^ and Amaral et al.^[16]^. According to the literature, supraventricular arrhythmias represent the main complications of adults with CHD and are the result of significant residual lesions, surgical scars, and depend on the degree of heart complexity and age at repair^[4,6,7,23]^.

Severe PAH with Eisenmenger syndrome was a frequent complication in patients with unrepaired high pulmonary blood flow defects, particularly ASD, and its frequency was higher than the reports in the literature^[6-7,25]^. Differently, in the study by Amaral et al.^[16]^, Eisenmenger syndrome was more frequently found in patients with unrepaired VSD. It is a serious illness and there are multiple factors involved in its etiology as the location of the shunt defect, genetic predisposition, and age^[6,7,19,25]^. So, it is possible that late diagnosis may have influenced the development of severe PAH and Eisenmenger syndrome in a meaningful parcel of the adult population with unrepaired high pulmonary blood flow defects studied herein, leading to obscure prognosis for these patients.

Briefly, the present study revealed a problem that requires substantial consideration, represented by high frequency of patients with unrepaired defects, predominantly ASD, as a consequence of late diagnosis. It is important to highlight that untreated children are future adults to be treated, as long as they are still feasible of treatment. Early diagnosis with invasive treatment is fundamental to avoid future complications and increase in morbimortality.

### Limitations

The limitations of the present research were its retrospective cross-sectional design and the lack of comparison between repaired and unrepaired patients in the clinical profile.

## CONCLUSION

The profile of adults with CHD in the present study revealed predominantly young adults, mainly women, coming from Fortaleza and the countryside in equal proportion. Acyanotic heart defects were the most frequent ones. Most patients had CHD of moderate complexity while heart disease of great complexity made up the minority of the cohort. *Ostium secundum* ASD was the most frequent defect followed by VSD and tetralogy of Fallot. Although more than half of the cohort had been repaired, unrepaired patients due to late diagnosis were a frequent finding that calls attention for a serious problem. Late diagnosis collaborated with the development of complications like arrhythmias, heart failure, and severe PAH, indicating the need for pursuing CHD diagnosis and repair in childhood.

## Abbreviations, Acronyms & Symbols

AF: Atrial fibrillation
AR: Aortic regurgitation
ASD: Atrial septal defect
BH: Blalock-Hanlon
CAVC: Complete atrioventricular canal
ccTGA: Congenitally corrected transposition of the great arteries
CHD: Congenital heart disease
CI: Confidence interval
CL: Confidence limit
DORV: Double outlet right ventricle
ECG: Electrocardiogram
FC: Functional class
IL: Inferior limit
IQI: Interquartile interval
MBTS: Modified Blalock-Taussig shunt
NSVT: Non-sustained ventricular tachycardia
NYHA: New York Heart Association
OP: *Ostium primum*
OS: *Ostium secundum*
PA: Pulmonary atresia
PAB: Pulmonary artery banding
PAH: Pulmonary arterial hypertension
PAPVC: Partial anomalous pulmonary venous connection
PDA: Patent *ductus arteriosus*
PS: Pulmonary stenosis
SD: Standard deviation
SL: Superior limit
SV: *Sinus venosus*
TAPVC: Total anomalous pulmonary venous connection
TGA: Transposition of the great arteries
VSD: Ventricular septal defect
